# Forming a choir with total laryngectomized patients

**DOI:** 10.1590/2317-1782/e20250122en

**Published:** 2026-04-07

**Authors:** Juliana Ceglio Monteiro, Sabrina Mazzer Paes, Renata Rangel Azevedo

**Affiliations:** 1 Departamento de Fonoaudiologia, Universidade Federal de São Paulo – UNIFESP - São Paulo (SP), Brasil.

**Keywords:** Singing, Speech, Language and Hearing Sciences, Laryngectomy, Rehabilitation, Esophageal Voice

## Abstract

This study describes the experience of forming a choir with patients who have undergone total laryngectomy. The choir began its activities in 2019 and is composed of total laryngectomy patients who are currently undergoing or have previously undergone therapy at the service and, therefore, have heterogeneous esophageal voice (EV) production. Thus, patients are subdivided into four groups, according to their level of EV acquisition: Group 1 – fluent in oral communication with EV and users of electrolarynx (perform chorus and solos); Group 2 – produce words with EV (perform final sections of verses); Group 3 – patients who produce syllables and vowels with EV (mark the rhythm with sequential syllable emission); Group 4 – produce no sound with EV (perform body percussion effects). All actively participate in selecting the repertoire. The choir also includes laryngeal voices (university students and speech-language-hearing pathologists). Rehearsals take place weekly, as do the therapy sessions. Musical arrangements must be adapted so that all patients can effectively participate in the group's overall sound. This activity fosters greater interaction among participants, strengthening emotional bonds, providing socialization, and sharing experiences beyond rehabilitation. The musical repertoire is carefully selected, considering the length of the verses, the tempo of the songs, the sustain of notes, and the melodic line.

## INTRODUCTION

Patients diagnosed with advanced laryngeal cancer undergo a challenging treatment and rehabilitation process, requiring the support of a multidisciplinary professional team with a head and neck surgeon, oncologist, psychologist, speech-language-hearing (SLH) pathologist, nutritionist, dentist, physiotherapist, and so forth^(1,2)^. Moreover, depending on the characteristics of the tumor and the patients' health conditions, some of them need to undergo a surgical procedure called total laryngectomy, completely removing the laryngeal framework. Thus, patients face significant difficulties in communicating, as voice and speech suffer a major negative impact^(1)^.

The literature describes some speech rehabilitation options for total laryngectomized patients, such as the acquisition of esophageal voice (EV), the use of an electrolarynx (EL), and the use of a tracheoesophageal prosthesis for EV emission – the latter being rather expensive, although considered the gold standard in the rehabilitation of these cases^(2,3)^. The assertive choice of rehabilitation method depends on each patient’s physical, anatomical, and financial conditions and must be analyzed individually, as each has advantages and disadvantages^(2,3)^. Regardless of the option chosen, patients must be included in SLH therapy and often face the long challenge of resuming communication through speech.

SLH rehabilitation has explored some therapeutic approaches, such as forming choirs with total laryngectomized patients.

Several choirs of total laryngectomy patients are active in different regions and services in Brazil. The José Alencar Gomes da Silva National Cancer Institute (INCA) was the first institution to create a choir of laryngectomy patients in the country in 1993, called INCAnto. Since then, various institutions have formed choirs with their laryngectomy patients, such as the Sua Voz Choir, from the A.C. Camargo Cancer Center in São Paulo, SP, the Amigos da Voz Choir, from the Cancer Institute of the State of São Paulo (ICESP) in São Paulo, SP, and many others, totaling 19 groups in Brazil^(4)^.

These data were presented in a survey by the Voice Department of the Brazilian SLH Society (SBFa) in the second semester of 2018^(4)^, but they do not represent the totality of groups currently existing in Brazil. New groups have emerged since then, such as the Coral Clarear, from the Federal University of São Paulo (UNIFESP) in São Paulo, SP, the subject of this study, which began its activities in 2019.

Naturally, singing is importantly different from speaking in terms of posture, breathing, vocal projection, pauses, and frequency modulation^(5)^. Singing also requires refined vocal production control, such as pitch, note sustain, stronger and weaker emissions in the music tempo, respecting its melody, harmony, and rhythm^(6)^. Therefore, singing can pose greater challenges than speech for total laryngectomized patients, serving as a strategy to optimize their communication rehabilitation.

Furthermore, the literature highlights the benefits of choral singing for the mental and physical health of its members, both in the general population and in patients undergoing treatment after a cancer diagnosis, including laryngeal cancer. This activity can help them interact socially and share experiences, contributing to their treatment and rehabilitation^(7)^. Total laryngectomized patients’ participation in choirs can also be beneficial to address their increased risk of anxiety, depression, and social isolation^(8)^.

Besides choirs, other types of support groups spread throughout Brazil are important therapeutic tools for patients undergoing rehabilitation. They offer emotional and social support, guidance on alternative communication and pulmonary rehabilitation, management of tracheostomy and tracheoesophageal prosthesis (when applicable), and information on social and pension rights^(9,10)^.

At first, a simplistic glance at choirs may suggest that it would be sufficient to gather the patients, provide them with an instrumental base, and ask them to sing some songs. However, in the case of total laryngectomy patients who do not yet emit EV or who only emit vowels, syllables, or isolated words through EV, musical arrangements must be adapted so that all patients can participate in the group and perceive their effective contribution to the collective sound result. Nonetheless, the literature lacks reports about choirs formed with total laryngectomy patients.

Therefore, this article aimed to describe the experience of forming a choir with patients who have undergone total laryngectomy.

## CLINICAL CASE PRESENTATION

This study took place in an SLH rehabilitation clinic linked to a university in the State of São Paulo, Brazil. As this is a descriptive report focusing on the strategies used, it is exempt from approval by the Research Ethics Committee and the application of an informed consent form, as provided for in Resolution No. 510/2016 of the Brazilian National Health Council. All participants signed a photo consent form.

This singing group began its activities in February 2019, still without a defined name, and its first public performance was at a scientific event of the institution in October of the same year. Later, in September 2022, the group was named Coral Clarear as a tribute to SLH pathologist Clara Rocha.

The group is composed of total laryngectomized patients who are in therapy at the institution's SLH rehabilitation service. It also includes patients who have already been discharged from SLH therapy but remain active in the choir. Students in their fourth year of the SLH undergraduate program and postgraduate students from the university also participate. Moreover, a SLH pathologist specializing in voice and the professor responsible for the service (public servants of the university) participate, managing the choir’s activities (rehearsals, performances, equipment, costumes, and sponsorships).

The patients who participate or have participated in the choir communicate orally through EV, regardless of their level of EV acquisition. Some of them use EL as an adjunct to EV acquisition. Patients using tracheoesophageal prostheses seldom participate.

Weekly 45-minute rehearsals take place in a consultation room at the SLH rehabilitation clinic. Following the rehearsals, patients undergoing rehabilitation attend individual 40-minute therapy sessions.

These patients’ oral communication rehabilitation combines techniques that require persistence. They generally aim at reducing possible edema in the neck region, relaxing and stretching the muscles of the shoulder girdle, improving the tone and mobility of the speech organs, improving intraoral proprioception, improving articulatory precision (fundamental for speech intelligibility with both EV and EL), and training visual communication acquisition with specific exercises.

It is difficult to learn to produce EV without a tracheoesophageal prosthesis, and the literature points to low success rates^(11)^. Nonetheless, it offers good cost-effectiveness, with no maintenance costs or risk of rejection, resulting in functional vocal quality. It is the most used in developing countries, such as Brazil^(12)^. EL can be a viable alternative for individuals who cannot achieve acceptable quality in EV production. Although EL does not restore natural vocal quality, it provides good intelligibility and is usually easy to learn, being especially indicated in cases with anatomical, motor, or cognitive limitations that make EV unfeasible.

Many of the therapeutic strategies mentioned above are worked on with the choir during rehearsals and are subsequently reinforced and personalized in individual therapy sessions, which take place afterward. It is important to emphasize that even though it is a group activity, it respects individual limitations, pace, and particularities.

A choir is composed of three or more people who can be divided into subgroups called voice types. This division is commonly carried out considering the expected tonalities in each melody, with a basic division of four voice types: basses and tenors, altos and sopranos^(13)^. However, EV frequency modulation is limited. Patients undergoing SLH therapy who participate in the choir have heterogeneous EV performance. Thus, the voice types were divided considering the EV acquisition levels of Wepman et al.^(14)^, a 7-point scale in which 7 is the worst classification (absence of EV production) and 1 is the best (fluent speakers, with automatic EV production)^(14)^.

Thus, the patients were divided into four subgroups:

- Group 1: composed of the best EV speakers (levels 1 and 2 of Wepman et al.^(14)^) and patients who use EL – i.e., those who can produce longer phrases, with some variation in frequency and intensity. This group performs the chorus and solos (musical passages performed by only one voice, the soloist).
- Group 2: composed of patients who produce some voluntary EV emissions, such as monosyllables and other words (levels 3 and 4 of Wepman et al.^(14)^). This group performs the chorus (although maintaining articulate speech most of the time) and the final sections of musical verses.
- Group 3: composed of patients who emit some EV sounds, but still involuntarily most of the time (levels 5 and 6 of Wepman et al.^(14)^). This group is focused on emitting sequential syllables, marking the rhythm at strategic moments in the music.
  - Group 4: composed of patients who do not emit any EV sound and do not use EL (level 7 of Wepman et al.^(14)^). This last group performs body percussion effects with lip clicks, tongue clicks, clapping, finger snaps, and so on.

The choir also includes laryngeal voices (all the other members mentioned above who are not laryngectomized patients), who contribute by sustaining the melody and assisting in moments when the music requires longer notes and greater transitions between low and high notes. Moreover, they assist as sub-conductors of the patients' groups at specific times, and students who can produce good EV and use EL are encouraged to sing in Group 1 along with the patients.

The songs the choir sings are selected according to the patients’ musical taste, limitations, and difficulties within the therapeutic process. Hence, at the beginning of each year, patients are asked about the songs they would like to rehearse in the choir. Then, the conductor selects the songs together with the patients, considering the length of the verses and the tempo of the music.

In addition to presentations at the university's in-person and online scientific events, the choir performs at the outpatient clinic in the middle of the year to support Green July (a national awareness campaign about head and neck cancer in Brazil) and at the end of each year, with a Christmas repertoire, as a finale of that year's activities. Besides the musical performance, the events include a space for fellowship and conversation, in which participants are encouraged to share feelings, challenges, and progress made throughout the treatment.

It is worth noting that to date, there is no self-assessment questionnaire on quality of life validated in Brazil aimed exclusively at laryngectomized patients who are part of choirs. Therefore, the benefits of this activity are analyzed through the patients' reports.

## DISCUSSION

Although challenging, it is possible to form a choir with patients who have undergone total laryngectomy. Adaptations are necessary for them to contribute effectively to the sound outcome, when compared to traditional choirs formed with laryngeal voices.

The first major challenge in singing for total laryngectomy patients is the difficulty in varying frequencies (high and low), which is what maintains the melodic line of the music. For laryngeal voices, this stems from changes in mass and tension in the vocal folds, something restricted in the pharyngo-esophageal segment, where EV is produced. Given these limitations, patients are taught and shown the importance of the concept of pitch (the sensation of frequency resulting from different adjustments in the positioning of the articulators of the vocal tract)^(15)^, starting with awareness strategies that include clapping with straighter hands (higher pitch) and clapping with cupped hands (lower pitch) or tongue clicking associated with a smile (higher pitch) and tongue clicking associated with a pout (lower pitch), marking the rhythm of the music, moving on to speech (with strategies planned in individual therapy) and singing. Patients in Group 1 are quite challenged in this regard: they must train their vocal range by singing the verses of the songs with good articulatory amplitude, refining the intensity and pitch to improve vocal expressiveness.

The greatest challenge for Groups 2 and 3 is the final emission of the musical verses (Group 2) and the repetition of syllables (Group 3) within the song’s rhythm. At times, patients produce mouth clicks (only the articulatory sound) instead of the EV. Nevertheless, by attempting to respect the rhythm of the music, patients report improvement in their ability to inject air into the pharyngo-esophageal segment, which is necessary for producing EV.

As for Group 4, performing percussion effects with sequential movements of the oral musculature (tongue click with a pout and smile, kiss with protracted lips, and kiss with retracted lips) serves as an articulatory exercise. Articulation is rather important for these patients, who communicate orally through articulated speech, while they do not acquire EV. It is also an exercise to improve the tone and mobility of the oral musculature, something crucial for developing EV^(16,17)^. These bring noticeable positive results in the patients’ clinical evolution, and they report preferring oral motor exercises at home in this way, with music.

The rehearsals always begin with stretching and relaxation of the shoulder girdle muscles, with instrumental music already playing in the background. Then the conductor selects a strategy to work on a basic musical element: 1- Melody (sequence of notes with differences in pitch – low and high, intensity – loud and soft, and duration – long and short); 2- Harmony (a sequence of simultaneous notes that make up a harmonic field – chords); 3- Rhythm (dictates the time of the notes and the music – measures)^(6)^. Understanding these basic concepts is important for patients and students who have never had prior experience with music. Finally, the group rehearses the songs they had selected, practicing with each group or participant individually and then all together. The goal is for all participants to experience the process and have their moment to shine, whether through rhythm or solo. Patients may go through different stages as they progress weekly within therapy; therefore, their role in the choir can change.

The undergraduate SLH students who participate in the choir are the pathologists responsible for the care of laryngectomized patients. Thus, they are encouraged to include strategies with the vocal emissions required in the choir's songs in their therapeutic plans.

Other strategies that contribute to EV acquisition are included individually, including breathing exercises, stretching, and postural exercises^(1,16,17)^.

The fact that the choir is invited to perform at scientific events at the university sets a deadline for doing the tasks required by the conductor properly, which motivates patients to intensify their training at home. In 2022, for example, the performance took place at an online scientific event and can be viewed on an internet video platform^(18)^.

The choice of music, considering its tempo and the length of the verses, is important for this group. Very long verses and fast tempo, with few intervals between verses, usually make it difficult to produce EV, and the patients request not to include such songs in the repertoire. Moreover, songs with sustained notes and melodic lines with a very wide range of low and high frequencies should be avoided.

As mentioned earlier, the postoperative period of total laryngectomy depends on a multidisciplinary team, and oral communication rehabilitation is challenging. These patients may face depression, anxiety, and social isolation during this period^(8)^. The choir activity provided greater interaction among the members, strengthening the patients’ bond with the pathologists and one another, exchanging contacts and sharing experiences beyond the outpatient clinic and the rehabilitation process.

Organizing a group project like this, of course, brings setbacks. Some patients sometimes need to be absent from rehearsals for a few weeks because they are unwell or because they are undergoing other more urgent or priority parallel treatments; others stop participating due to their death; most patients who are discharged, who have good vocal production (the good speakers) and make up Group 1, stop attending the choir because they do not have the financial means or availability to participate in weekly rehearsals (currently, only one patient returns to rehearsals weeks before a performance); undergraduate students (interns) cannot participate in rehearsals throughout the academic year, as they rotate every 3 months, requiring the conductor to review some musical concepts and rehearse some songs with each group of new students; also, with this turnover, few students acquire good vocal production to compose Group 1, which sustains the melody. Thus, the choir depends on laryngeal voices to assist in its final sound result and relies on volunteer conductor and instrumentalists. The latter is usually the one with musical training, being responsible for classifying the singers according to their voice groups and conducting rehearsals and performances^(19,20)^. Currently, a postgraduate SLH pathologist with experience as a singing teacher conducts this choir, a patient from Group 1, who has already been discharged, sings and plays bass in some performances, and occasionally students or patients who play a musical instrument join the group with their skills.

Finally, it is known in the literature that group activities can assist in these patients’ therapeutic process^(9,10)^. In the case of this experience report, the dialogue space held at the end of each school year strengthens the bond between the members, stimulates mutual support, and values ​​individual progress. This consolidates the annual closing as a significant moment in the rehabilitation process and promotion of the well-being of the group members. Hence, including choral singing in the rehabilitation of total laryngectomy patients adds positive results: the repetition of exercises in the traditional session increases, the activity allows patients to put these training exercises into practice more frequently in their daily lives, and patients report that the exercises are performed more enjoyably and promote a feeling of well-being. This activity also benefits patients in the initial therapeutic process, who do not yet produce EV but can still actively participate in the choir, carrying out strategies that are independent of EV emission. Patients commonly report a preference for performing articulation exercises, improving tone and mobility of the orofacial muscles, and training to acquire EV when combined with music, which contributes to greater adherence to therapy.

## FINAL COMMENTS

Choral singing can be adapted for individuals with heterogeneous oral emission, obtaining benefits in both communication and socialization. This activity can be adapted so that all total laryngectomized patients can participate, regardless of their level of EV acquisition or organic limitations.

The experience of forming this choir teaches that the careful choice of musical repertoire is crucial and should consider the length of the verses and the tempo of the songs. Moreover, songs that require sustained notes and that have a melodic line with a wide range of low and high notes should be avoided or adapted.

## References

[1] Clarke P, Radford K, Coffey M, Stewart M (2016). Speech and swallow rehabilitation in head and neck cancer: United Kingdom National Multidisciplinary Guidelines. J Laryngol Rhinol Otol.

[2] Egan MY, McEwen S, Sikora L, Chasen M, Fitch M, Eldred S (2013). Rehabilitation following cancer treatment. Disabil Rehabil.

[3] Ahlberg A, Engström T, Nikolaidis P, Gunnarsson K, Johansson H, Sharp L (2011). Early self-care rehabilitation of head and neck cancer patients. Acta Otolaryngol.

[4] Rossi VC, Guimarães MF, Moreira MJS, Lopes L, Moreti F (2020). Corais de indivíduos laringectomizados totais no Brasil. CoDAS.

[5] Behlau M, Rehder MI (2009). Higiene vocal: para o canto coral..

[6] Med B (1996). Teoria da música..

[7] Moss H, Lynch J, O’Donoghue J (2018). Exploring the perceived health benefits of singing in a choir: an international cross-sectional mixed-methods study. Perspect Public Health.

[8] Offerman MPJ, Pruyn JFA, de Boer MF, Busschbach JJV, de Jong RJB (2015). Psychosocial consequences for partners of patients after total laryngectomy and for the relationship between patients and partners. Oral Oncol.

[9] ACBG Brasil: Associação Brasileira de Câncer de Cabeça e Pescoço (2025). Inclusão.

[10] Ribeiro VV, Panhoca I, Dassie-Leite AP, Bagarollo MF (2012). Grupo terapêutico em fonoaudiologia: revisão de literatura. Rev CEFAC.

[11] Raquel ACS (2018). Proficiência da voz esofágica e qualidade de vida em laringectomizados totais.

[12] Xi S (2010). Effectiveness of voice rehabilitation on vocalisation in postlaryngectomy patients: a systematic review. Int J Evid-Based Healthc.

[13] Fernandes AJ (2009). O regente e a construção da sonoridade coral: uma metodologia de preparo vocal para coros.

[14] Wepman JM, MacGahan JA, Rickard JC, Shelton NW (1953). The objective measurement of progressive esophageal speech development. J Speech Hear Disord.

[15] Sundberg J, Titze I, Scherer R (1993). Phonatory control in male singing: A study of the effects of subglottal pressure, fundamental frequency, and mode of phonation on the voice source. J Voice.

[16] Sahin M, Vardar R, Kirazli T, Ogut F, Akyildiz S, Bor S (2015). Predictive value of esophageal motility test in the proficiency of esophageal speech. Dis Esophagus.

[17] Martin DE, Hoops HR, Shanks JC (1974). The relationship between esophageal speech proficiency and selected measures of auditory function. J Speech Hear Res.

[18] Paes SFGA (2025). Coral Clarear - SIJO Unifesp 2022.

[19] Ramos MAS (1988). Canto coral: do repertório temático à construção do programa.

[20] DiMartino D (1998). Music in the 20th century..

