# Supplementary material for: Forming a choir with total laryngectomized patients
Source: Codas. 2026 Apr 7;38(3):e20250122. doi: 10.1590/2317-1782/e20250122en (PMC13143028; doi:10.1590/2317-1782/e20250122en)
Supplement: Translation

# Translation: Formação de grupo de canto coral com pacientes submetidos à laringectomia total

**DOI:** 10.1590/2317-1782/e20250122pt

**Authors:** Juliana Ceglio Monteiro, Sabrina Mazzer Paes, Renata Rangel Azevedo

**Affiliations:** 1 Departamento de Fonoaudiologia, Universidade Federal de São Paulo – UNIFESP - São Paulo (SP), Brasil.

**Keywords:** Canto, Fonoaudiologia, Laringectomia, Reabilitação, Voz Esofágica,

## Abstract

Descrever a experiência no processo de formação de um grupo de canto coral com pacientes submetidos à laringectomia total. Este grupo de canto iniciou suas atividades em 2019 e é composto por pacientes laringectomizados totais que estão ou que já estiveram em terapia no serviço e, portanto, apresentam desempenho heterogêneo quanto à emissão da voz esofágica (VE). Por este motivo, os pacientes são subdivididos em quatro naipes, de acordo com o nível de aquisição de VE que se encontram: Naipe 1 – fluentes na comunicação oral com a VE e usuários de laringe eletrônica (realizam coros e solos); Naipe 2 – produzem palavras com a VE (realizam trechos finais dos versos); Naipe 3 – pacientes que emitem sílabas e vogais com a VE (realizam a marcação rítmica com emissão de sílabas sequenciais); Naipe 4 – não emitem som algum com a VE (realizam efeitos percussivos corporais). Todos participam ativamente da escolha do repertório. O coral também é composto por vozes laríngeas (alunos da Universidade e fonoaudiólogos). Os ensaios acontecem semanalmente, bem como as sessões de terapia. É preciso adaptar os arranjos musicais para que todos os pacientes possam participar efetivamente do resultado sonoro do grupo. Esta atividade proporciona maior interação entre os participantes, fortalecendo o vínculo afetivo, a socialização e permitindo o compartilhamento de experiências para além do processo de reabilitação. É preciso escolher cuidadosamente o repertório musical, considerando a extensão dos versos, o andamento das músicas, a sustentação de notas e a linha melódica.

## INTRODUÇÃO

Os pacientes diagnosticados com câncer de laringe avançado passam por um processo de tratamento e de reabilitação desafiador, que exige o apoio de uma equipe profissional multidisciplinar composta por médico cirurgião de cabeça e pescoço, oncologista, psicólogo, fonoaudiólogo, nutricionista, dentista, fisioterapeuta, entre outros^(1,2)^. Além disso, dependendo das características do tumor e das condições de saúde dos pacientes, alguns deles precisam ser submetidos a um procedimento cirúrgico chamado laringectomia total, que resulta na remoção completa do arcabouço laríngeo. Com a retirada dessa estrutura, os pacientes enfrentam dificuldades importantes para se comunicar, já que a voz e a fala sofrem grande impacto negativo^(1)^.

Existem algumas opções de reabilitação da fala descritas na literatura para a população de pacientes laringectomizados totais, como a aquisição da voz esofágica (VE), o uso de uma laringe eletrônica (LE) e o uso da prótese traqueoesofágica para a emissão da VE, sendo que esta última opção, embora considerada padrão-ouro na reabilitação destes casos, apresenta alto custo^(2,3)^. A escolha assertiva do método de reabilitação depende das condições físicas, anatômicas e financeiras de cada paciente e devem ser analisadas de maneira individual, pois cada uma tem vantagens e desvantagens^(2,3)^. Independentemente da opção escolhida, os pacientes devem ser inseridos em terapia fonoaudiológica e enfrentam, na maioria das vezes, um longo desafio: o de voltar a se comunicar através da fala.

No contexto da reabilitação fonoaudiológica, algumas abordagens terapêuticas têm sido exploradas, como é o caso da formação de grupos de canto coral com pacientes laringectomizados totais.

Há diversos grupos de canto coral compostos por laringectomizados totais atuantes em diferentes regiões e serviços no Brasil, tendo sido o Instituto Nacional de Câncer José Alencar Gomes da Silva (INCA) a primeira instituição a criar o coral de laringectomizados no país, em 1993, denominado INCAnto. Desde então, diversas instituições passaram a formar coros com seus pacientes laringectomizados, como é o caso do Coral Sua Voz, do A.C. Camargo Cancer Center em São Paulo, SP, o Coral Amigos da Voz, do Instituto do Câncer do Estado de São Paulo (ICESP) em São Paulo, SP, dentre muitos outros, totalizando 19 grupos no Brasil^(4)^.

Esses dados foram apresentados em um levantamento realizado pelo Departamento de Voz da Sociedade Brasileira de Fonoaudiologia (SBFa) no segundo semestre de 2018^(4)^ e não representam a totalidade dos grupos existentes no Brasil atualmente, visto que surgiram novos grupos desde então, como é o caso do Coral Clarear, da Universidade Federal de São Paulo (UNIFESP) em São Paulo, SP, objeto desse estudo, que iniciou suas atividades em 2019.

Naturalmente, existem diferenças importantes entre a produção da fala e do canto observadas na postura, na respiração, na projeção vocal, no uso de pausas e na modulação de frequências^(5)^. Além disso, o canto exige controles refinados da produção vocal, como afinação, sustentação de notas, emissões mais fortes e mais fracas, no tempo exigido pela música, respeitando sua melodia, a harmonia e o ritmo^(6)^. Portanto, o canto pode ser considerado ainda mais desafiador que a fala para os pacientes laringectomizados totais, podendo servir como estratégia de otimização do processo de reabilitação da comunicação para esta população.

Além disso, a literatura destaca os benefícios do canto coral para a saúde mental e física de seus integrantes, tanto na população em geral, como em pacientes em tratamento após algum diagnóstico de câncer, incluindo os de laringe. Essa atividade pode promover a interação social, possibilitar o compartilhamento de experiências e, consequentemente, contribuir para o processo de tratamento e de reabilitação^(7)^ e, considerando o aumento do risco de ansiedade, depressão e isolamento social em pacientes submetidos à laringectomia total^(8)^, a participação em grupos de canto coral pode ser benéfica.

Além dos grupos de canto coral, existem outros tipos de grupos de apoio disseminados pelo Brasil e que configuram ferramentas terapêuticas importantes para os pacientes em processo de reabilitação, oferecendo acolhimento emocional e social, orientações sobre comunicação alternativa, orientações sobre a reabilitação pulmonar, o manejo com a traqueostomia e com a prótese traqueoesofágica (quando é o caso), além de oferecer informações sobre direitos sociais e previdenciários^(9,10)^.

A princípio, de maneira simplista, quando se pensa em um grupo de canto coral pode parecer suficiente reunir os pacientes, fornecer-lhes uma base instrumental e solicitar que cantem algumas músicas. No entanto, ao considerar pacientes laringectomizados totais que ainda não emitem VE ou que emitem apenas vogais, sílabas ou palavras isoladas por meio da VE, torna-se imperativo elaborar arranjos musicais adaptados, de modo que todos os pacientes possam participar do grupo e perceber sua efetiva contribuição para o resultado sonoro do coletivo. Contudo, faltam relatos na literatura acerca da formação de grupos de canto coral com pacientes laringectomizados totais.

Portanto, este artigo tem como objetivo descrever a experiência no processo de formação de um grupo de canto coral com pacientes submetidos à laringectomia total.

## APRESENTAÇÃO DO CASO CLÍNICO

O presente estudo ocorreu em um ambulatório de reabilitação fonoaudiológica ligado a uma Universidade no Estado de São Paulo, Brasil. Por se tratar de um relato descritivo e com foco nas estratégias utilizadas, este trabalho está isento de aprovação pelo Comitê de Ética em Pesquisa bem como a aplicação do Termo de Consentimento Livre e Esclarecido, conforme previsto na Resolução nº 510/2016 do Conselho Nacional de Saúde. Todos os participantes assinaram o Termo de Autorização de Exibição de Imagens.

Este grupo de canto iniciou suas atividades em fevereiro de 2019, ainda sem nome definido, e sua primeira apresentação em público foi em um evento científico da própria instituição, em outubro do mesmo ano. Posteriormente, em setembro de 2022, como homenagem à Fga. Clara Rocha, este grupo de canto foi denominado de Coral Clarear.

O grupo é composto pelos pacientes laringectomizados totais que estão em terapia no serviço de reabilitação fonoaudiológica da instituição, contando, inclusive, com a participação de pacientes que já receberam alta da terapia fonoaudiológica e mantêm-se ativos no coral. Participam também alunos do 4° ano do curso de graduação em Fonoaudiologia, além de alunos da pós-graduação da Universidade. Além disso, participam uma fonoaudióloga especialista em voz e a docente responsável pelo serviço (servidoras públicas da Universidade) que administram as atividades desse grupo de canto (ensaios, apresentações, equipamentos, figurinos e patrocínios).

Os pacientes que participam ou que já participaram desse grupo de canto coral comunicam-se oralmente através da VE, independentemente do nível de aquisição de VE em que se encontram; alguns deles fazem uso de LE como coadjuvante à aquisição da VE; não é comum a presença de pacientes em uso de prótese traqueoesofágica.

Os ensaios acontecem semanalmente em uma sala de atendimento do próprio ambulatório de reabilitação fonoaudiológica e tem duração de 45 minutos. Na sequência dos ensaios, os pacientes que estão no processo de reabilitação frequentam a sessão de terapia individual, com duração de 40 minutos.

A reabilitação da comunicação oral destes pacientes envolve uma combinação de técnicas que requer persistência. Geralmente abrangem técnicas que visam reduzir possíveis edemas na região do pescoço, relaxar e alongar a musculatura da cintura escapular, melhorar o tônus e a mobilidade dos órgãos fonoarticulatórios, melhorar a propriocepção intraoral, melhorar a precisão articulatória (fundamental para a inteligibilidade da fala com a VE e com a LE), bem como exercícios específicos do treino de aquisição da VE.

A VE produzida sem uma prótese traqueoesofágica, apesar de ser de difícil aprendizagem e a literatura apontar baixas taxas de sucesso^(11)^, apresenta bom custo-benefício, visto que não há necessidade de gastos com manutenção, não apresenta riscos de rejeição e resulta em uma qualidade vocal funcional, sendo a mais utilizada em países em desenvolvimento, como é o caso do Brasil^(12)^. Para os indivíduos que não conseguem atingir uma qualidade aceitável na produção da VE, a LE pode ser uma alternativa viável. Embora a laringe eletrônica não restabeleça a qualidade vocal natural, ela proporciona boa inteligibilidade e costuma ser de fácil aprendizado, sendo especialmente indicada em casos com limitações anatômicas, motoras ou cognitivas que inviabilizam a VE.

Muitas das estratégias terapêuticas mencionadas acima são trabalhadas com o grupo de canto coral durante os ensaios e, posteriormente, são reforçadas e personalizadas nas sessões individuais de terapia, que acontecem na sequência. É importante ressaltar que mesmo sendo uma atividade em grupo, as limitações individuais, o ritmo e as particularidades de cada paciente são respeitados.

Um coral é composto por três ou mais pessoas que podem ser divididas em subgrupos chamados de naipes. Essa divisão é comumente realizada considerando as tonalidades esperadas em cada melodia, tendo como divisão básica quatro naipes: baixos e tenores, contraltos e sopranos^(13)^. Contudo, a modulação de frequências na emissão da VE é limitada. Os pacientes que estão em terapia fonoaudiológica, que participam do coral, apresentam desempenho heterogêneo quanto à emissão da VE. Desta forma, a divisão de naipes foi realizada considerando os níveis de aquisição de VE de Wepman et al.^(14)^, uma escala de sete pontos, sendo sete a pior classificação (ausência de produção de VE) e um a melhor (falantes fluentes, com produção de VE automática)^(14)^.

Portanto, os pacientes foram divididos em quatro subgrupos:

- Naipe 1: composto pelos melhores falantes de VE (níveis 1 e 2 de Wepman et al.^(14)^) e pelos pacientes que fazem uso de LE, ou seja, por aqueles que estão aptos a produzir frases mais longas, com certa variação de frequência e intensidade. Este grupo é direcionado para a execução dos coros e dos solos (trecho musical executado por apenas uma voz, o solista);
- Naipe 2: composto pelos pacientes que produzem algumas emissões voluntárias com a VE, como monossílabos e outras palavras (níveis 3 e 4 de Wepman et al.^(14)^). Este grupo é direcionado para a realização dos coros (ainda que mantenha fala articulada na maior parte do tempo) e para a emissão de trechos finais dos versos musicais;
- Naipe 3: composto pelos pacientes que emitem alguns sons com a VE, mas ainda de maneira involuntária na maior parte das vezes (níveis 5 e 6 de Wepman et al.^(14)^). Este grupo é direcionado para emitir sílabas sequenciais marcando o ritmo em momentos estratégicos da música;
  - Naipe 4: composto pelos pacientes que não emitem som algum com a VE e nem fazem uso de LE (nível 7 de Wepman et al.^(14)^). Esse último grupo é direcionado para realizar efeitos percussivos corporais com estalos de lábios, estalos de língua, palmas, estalos de dedos, entre outros.

Em paralelo, o grupo também é composto por vozes laríngeas (todos os outros integrantes mencionados acima que não são os pacientes laringectomizados), que contribuem sustentando a melodia e auxiliam nos momentos em que a música exige sustentação de notas mais longas e maior transição entre graves e agudos. Além disso, eles auxiliam como subregentes dos naipes dos pacientes em momentos específicos e os alunos que sabem produzir boa VE, assim como os que conseguem utilizar a LE, são estimulados a cantar no Naipe 1, juntamente com os pacientes.

As músicas a serem trabalhadas no coral são selecionadas levando-se em conta o gosto musical dos pacientes, bem como suas limitações e dificuldades dentro do processo terapêutico. Para tanto, todo início de ano os pacientes são questionados sobre as músicas que eles gostariam de ensaiar no coral. Depois disso, a regente seleciona as músicas juntos com os pacientes considerando a extensão dos versos e o andamento da música.

Além das apresentações nos eventos científicos da Universidade, sendo eles presenciais ou online, o coral realiza uma apresentação no ambulatório no meio do ano, como forma de apoio ao Julho Verde (uma campanha nacional de conscientização sobre o câncer de cabeça e pescoço no Brasil) e outra apresentação no final de cada ano, com repertório natalino, para marcar o encerramento das atividades daquele ano. Além da performance musical, promove-se um espaço de confraternização e de diálogo, no qual os participantes são incentivados a compartilhar sentimentos, desafios e avanços obtidos ao longo do tratamento.

Vale ressaltar que até o momento não há validado no Brasil nenhum questionário de autoavaliação sobre qualidade de vida voltado exclusivamente para pacientes laringectomizados que fazem parte de grupos de canto coral, portanto, os benefícios desta atividade são analisados através dos relatos dos pacientes.

## DISCUSSÃO

A formação de um grupo de canto coral com pacientes submetidos à laringectomia total, apesar de desafiadora, é possível e, para que eles possam contribuir de maneira efetiva com o resultado sonoro, há a necessidade de adaptações quando comparada à formação de grupo de canto coral tradicional, com vozes laríngeas.

O primeiro grande desafio no canto para os pacientes laringectomizados totais é a dificuldade de variação de frequências (agudos e graves), que é o que mantém a linha melódica da música. Para vozes laríngeas ela decorre de alterações de massa e tensão nas pregas vocais, algo restrito no segmento faringo-esofágico, onde a VE é produzida. Diante dessas limitações, o conceito de *pitch* (sensação de frequência decorrentes dos diferentes ajustes no posicionamento dos articuladores do trato vocal)^(15)^ é valorizado e ensinado aos pacientes, iniciando com estratégias de conscientização que inclui palmas com as mãos mais retas (som mais agudo) e palmas com as mãos em concha (som mais grave) ou estalo de língua associado a sorriso (som mais agudo) e estalo de língua associado a bico (som mais grave), fazendo a marcação rítmica da música, passando para a fala (com estratégias previstas na terapia individual) e para o canto. Os pacientes do Naipe 1 são bastante exigidos em relação a este quesito: eles devem treinar a VE cantando os versos das músicas com boa amplitude articulatória, refinando a intensidade e o *pitch* a fim de melhorar a expressividade vocal.

Para o Naipe 2 e 3, o maior desafio encontrado foi a emissão final dos versos musicais (Naipe 2) e a repetição de sílabas (Naipe 3) dentro do contexto rítmico exigido pela música. Em alguns momentos, os pacientes produzem cliques bucais (apenas o som articulatório) ao invés da VE. Apesar disso, com a tentativa de respeitar o ritmo da música, os pacientes relatam melhora na habilidade de injeção de ar no segmento faringo-esofágico, necessária para a produção da VE.

Para o Naipe 4, a realização dos efeitos percussivos com os movimentos sequenciais da musculatura oral (estalo de língua com bico e sorriso, beijo com os lábios protraídos e beijo com os lábios retraídos) serve como exercício articulatório, dada a importância da articulação para estes pacientes que se comunicam oralmente através da fala articulada, enquanto não adquirem a VE. Além disso, serve como exercício de melhoria de tônus e mobilidade da musculatura oral, algo crucial para o desenvolvimento da VE^(16,17)^, sendo possível observar resultados positivos na evolução clínica dos pacientes, além de eles relatarem preferência por realizar os exercícios de motricidade oral em casa desta forma, com música.

Os ensaios iniciam-se sempre com alongamento e relaxamento da musculatura da cintura escapular, já com a música instrumental de fundo. Em seguida a regente seleciona uma estratégia para trabalhar um elemento musical básico: 1- Melodia (sequência de notas com diferenças de altura – graves e agudas, intensidade – fortes e fracas, e duração – longas ou curtas); 2- Harmonia (encadeamento de notas simultâneas que compõem um campo harmônico – acordes); 3- Ritmo (dita o tempo das notas e da música – compassos)^(6)^. O entendimento desses conceitos básicos é importante para os pacientes e alunos que nunca tiveram experiência prévia com música. Por fim, as músicas selecionadas pelo grupo são ensaiadas, treinando com cada naipe ou participante individualmente e, em seguida todos juntos. O objetivo é que todos os participantes possam vivenciar a experiência e ter o seu momento de evidência seja através da rítmica ou do solo. Os pacientes podem passar por diferentes etapas, visto que a cada semana sua evolução dentro da terapia pode avançar e, portanto, o seu papel no coral pode ser modificado.

Os alunos da graduação do curso de fonoaudiologia que participam do coral são os terapeutas responsáveis pelos atendimentos dos laringectomizados. Com isso, eles são encorajados a incluir nos planos terapêuticos estratégias com as emissões vocais exigidas nas músicas do coral.

Cabe mencionar que outras estratégias que contribuem para a aquisição da VE são incluídas individualmente, entre elas exercícios de respiração, alongamentos e exercícios posturais^(1,16,17)^.

O fato de o coral ser convidado para se apresentar em eventos científicos da Universidade coloca prazo para a boa execução das tarefas exigidas pela regente, o que motiva os pacientes a intensificarem os treinos em casa. Em 2022, por exemplo, a apresentação aconteceu em um evento científico online e pode ser conferida em plataforma de vídeos na internet^(18)^.

A escolha das músicas é algo importante para este grupo, tendo que considerar a extensão dos versos e o andamento da música (versos muito longos e andamento rápido, com poucos intervalos entre os versos, costuma dificultar a emissão da VE e os próprios pacientes solicitam a não inclusão da música no repertório). Além disso, músicas com sustentação de notas e com linha melódica com alcance muito amplo de frequências graves e agudas devem ser evitadas.

Como dito anteriormente, a cirurgia de laringectomia total carrega consigo um pós-operatório que depende de uma equipe multiprofissional e o processo de reabilitação da comunicação oral é desafiador, podendo, nesse período, surgir quadros de depressão, ansiedade e isolamento social nessa população^(8)^. Durante esta atividade, foi observada maior interação entre os integrantes, fortalecendo o vínculo paciente-terapeuta e entre os pacientes que, por sua vez, trocam contatos e compartilham experiências para além do ambulatório e do processo de reabilitação.

A organização de um trabalho em grupo como este, evidentemente, traz contratempos: alguns pacientes por vezes precisam se ausentar dos ensaios por algumas semanas por não estarem dispostos ou por estarem em outros tratamentos paralelos mais urgentes ou prioritários; outros deixam de participar em virtude do seu falecimento; a maioria dos pacientes que recebe alta, que apresentam boa produção de VE (os bons falantes) e que compõem o Naipe 1, deixam de frequentar o coral por não terem condições financeiras ou disponibilidade para participarem semanalmente dos ensaios (atualmente, apenas um paciente volta a frequentar os ensaios semanas antes de alguma apresentação); os alunos da graduação (estagiários) não conseguem participar dos ensaios durante todo o ano letivo, pois eles rodam a cada três meses, tendo que a regente retomar alguns conceitos musicais e o ensaio de algumas músicas com cada grupo de novos alunos; além disso, com essa rotatividade, poucos alunos adquirem boa VE para compor o Naipe 1, que seria o naipe de sustentação da melodia. Com isso, o coral depende de vozes laríngeas para auxiliar no resultado sonoro final do coral; o coral depende de instrumentistas e de regente voluntários. O regente geralmente é o que possui formação musical, sendo o responsável pela classificação dos cantores de acordo com os naipes, além de conduzir os ensaios e as apresentações^(19,20)^. Atualmente uma pós-graduanda fonoaudióloga e com experiência em pedagogia do canto rege este coral, um paciente do Naipe 1, que já recebeu alta, canta e toca baixo em algumas apresentações e eventualmente surgem alunos ou pacientes que tocam algum instrumento musical e agregam o grupo com suas habilidades.

Por fim, é conhecido na literatura que os grupos podem auxiliar no processo terapêutico desse grupo de pacientes^(9,10)^, no caso do relato dessa experiência, o espaço de diálogo realizado no fim de cada ano letivo fortalece o vínculo entre os integrantes, estimula o apoio mútuo e valoriza o progresso individual. Desta forma, o encerramento anual consolida-se como um momento significativo no processo de reabilitação e promoção do bem-estar dos integrantes do grupo. É possível observar que a inclusão da atividade do canto coral inserida no processo de reabilitação de pacientes laringectomizados totais agrega resultados positivos: há um aumento da repetição dos exercícios somados na sessão tradicional, a atividade possibilita que os pacientes possam colocar em prática com maior frequência esses treinos no seu dia a dia, os pacientes relatam que os exercícios podem ser realizados de maneira mais prazerosa e que eles promovem sensação de bem-estar. Essa atividade favorece também os pacientes em processo terapêutico inicial, que ainda não produzem VE, mas que ainda assim podem participar ativamente do coral, realizando estratégias que independem de emissão de VE. É comum os pacientes relatarem preferência pela realização dos exercícios de articulação, de melhora do tônus e da mobilidade da musculatura orofacial e até mesmo do treino de aquisição de voz esofágica quando associados à música, o que contribui para maior adesão à terapia.

## COMENTÁRIOS FINAIS

É possível adaptar o canto coral para um grupo de indivíduos com emissão oral heterogênea e obter benefícios tanto na comunicação quanto na socialização. É possível adaptar esta atividade para que todos os pacientes laringectomizados totais possam participar, independentemente do seu nível de aquisição de VE ou mesmo diante das limitações orgânicas encontradas em cada caso.

A experiência com a formação deste grupo de canto coral ensina que a escolha criteriosa do repertório musical é crucial e deve considerar a extensão dos versos e o andamento das músicas. Cabe ressaltar que músicas que exigem sustentação de notas e que tenham linha melódica com amplo alcance de graves e agudos devem ser evitadas ou adaptadas.
